# mTOR signaling regulates central and peripheral circadian clock function

**DOI:** 10.1371/journal.pgen.1007369

**Published:** 2018-05-11

**Authors:** Chidambaram Ramanathan, Nimish D. Kathale, Dong Liu, Choogon Lee, David A. Freeman, John B. Hogenesch, Ruifeng Cao, Andrew C. Liu

**Affiliations:** 1 Department of Biological Sciences, University of Memphis, Memphis, Tennessee, United States of America; 2 Department of Biomedical Sciences, University of Minnesota Medical School, Duluth, Minnesota, United States of America; 3 Department of Biomedical Sciences, Program in Neuroscience, College of Medicine, Florida State University, Tallahassee, Florida, United States of America; 4 Divisions of Human Genetics and Immunobiology, Cincinnati Children's Hospital Medical Center, Cincinnati, Ohio, United States of America; 5 Department of Neuroscience, University of Minnesota Medical School, Minneapolis, Minnesota, United States of America; 6 Department of Physiology and Functional Genomics, University of Florida College of Medicine, Gainesville, Florida, United States of America; Charité - Universitätsmedizin Berlin, GERMANY

## Abstract

The circadian clock coordinates physiology and metabolism. mTOR (mammalian/mechanistic target of rapamycin) is a major intracellular sensor that integrates nutrient and energy status to regulate protein synthesis, metabolism, and cell growth. Previous studies have identified a key role for mTOR in regulating photic entrainment and synchrony of the central circadian clock in the suprachiasmatic nucleus (SCN). Given that mTOR activities exhibit robust circadian oscillations in a variety of tissues and cells including the SCN, here we continued to investigate the role of mTOR in orchestrating autonomous clock functions in central and peripheral circadian oscillators. Using a combination of genetic and pharmacological approaches we show that mTOR regulates intrinsic clock properties including period and amplitude. In peripheral clock models of hepatocytes and adipocytes, mTOR inhibition lengthens period and dampens amplitude, whereas mTOR activation shortens period and augments amplitude. Constitutive activation of mTOR in *Tsc2*^*–/–*^fibroblasts elevates levels of core clock proteins, including CRY1, BMAL1 and CLOCK. Serum stimulation induces CRY1 upregulation in fibroblasts in an mTOR-dependent but *Bmal1*- and *Period*-independent manner. Consistent with results from cellular clock models, mTOR perturbation also regulates period and amplitude in the *ex vivo* SCN and liver clocks. Further, mTOR heterozygous mice show lengthened circadian period of locomotor activity in both constant darkness and constant light. Together, these results support a significant role for mTOR in circadian timekeeping and in linking metabolic states to circadian clock functions.

## Author summary

The circadian clock coordinates daily physiology and metabolism in animals. There has been considerable interest in identifying mechanisms that link metabolic signals to circadian time keeping. mTOR (mammalian/mechanistic target of rapamycin) is a major intracellular sensor that integrates nutrient and energy status to fundamental cellular processes. Previous studies have identified a key role for mTOR in regulating photic entrainment and synchrony of the central circadian clock in the suprachiasmatic nucleus (SCN). Given that mTOR activities exhibit robust circadian rhythms in a variety of cells and tissues including the SCN, here we investigated the role of mTOR in orchestrating autonomous functions in central and peripheral circadian clocks. Using a combination of genetic and pharmacological approaches we show that mTOR inhibition slows down the circadian clock and dampens clock oscillations, whereas mTOR activation accelerates the clock and enhances clock oscillations in cells, tissues as well as in mice. Together, these results support a significant role for mTOR in linking metabolic states to circadian time keeping.

## Introduction

The circadian clock regulates the sleep/wake cycle and all associated cellular, metabolic and physiological processes in animals. Disruption of the circadian system is associated with a variety of disease states, including sleep disorders, metabolic syndromes, and cardiovascular diseases[1–3]. In mammals, the central clock is located in the hypothalamic suprachiasmatic nucleus (SCN). The SCN receives photic input from the intrinsically photosensitive retinal ganglion cells and relays the light/dark information to extra-SCN brain regions and peripheral tissues via neural and endocrine signals. In this manner, the SCN synchronizes a myriad of peripheral oscillators into a coherent time-keeping system[4]. At the molecular level, the circadian clock is based on a transcriptional negative feedback loop, in which the bHLH-PAS domain containing transcriptional activators BMAL1 and CLOCK form a heterodimeric complex to activate E-box cis element-mediated transcription of *Period* (*Per1*, *2*, *3*) and *Cryptochrome* (*Cry1*, *2*) genes. PER and CRY proteins are the repressor components that, upon translocation to the nucleus, suppress the transcriptional activity of BMAL1 and CLOCK[4,5]. This core loop regulates and intertwines with two interlocking loops mediated by the RRE and D-box elements. These molecular mechanisms underlie rhythmic expression of thousands of genes and consequently various cellular functions and processes[6,7].

While the genetic basis of circadian behavior has been well established, additional clock components exist[8] and new players have emerged in recent years[9–11]. Although there have been major advances in our understanding of circadian outputs and tissue/cell type-specific physiological functions, especially at the genome-wide level, the converse regulation is less understood, i.e. how the circadian clock is integrated with and regulated by the many processes that are under its own control. In an effort to identify additional clock components and modifiers, we carried out a genome-wide RNAi screen in a human U2OS cellular clock model and identified hundreds of genes whose knockdown impacted cellular clock function[12]. Pathway analysis revealed that these modifiers were members of many cellular pathways and functions, among which the insulin signaling pathway is the most overrepresented. Downregulation of multiple components of the insulin pathway resulted in period changes, e.g. PI3K (PIK3R5, long period) and mTOR (FRAP1, long period). Conversely, PI3K and mTOR are among the many pathway components that are regulated at the transcriptional level by the circadian clock[7,13]. These results highlight the functional interaction between insulin signaling and the circadian clock. Recent studies have uncovered several more examples of this interplay, especially with metabolism[14,15]. In the current study, we show how the mTOR pathway, a key metabolic regulator, modulates the circadian clock function.

The mammalian/mechanistic target of rapamycin (mTOR), also known as FK506-binding protein 12-rapamycin-associated protein 1 (FRAP1), is a highly conserved nutrient-activated Ser/Thr protein kinase that functions to sense and integrate with cellular metabolism and growth[16–19]. The upstream signals include growth factors such as insulin and insulin-like growth factor-1 (IGF-1), energy status such as ATP levels, and nutrient availability (e.g. leucine and arginine levels)[16–19]. In response to these extracellular and intracellular cues, mTOR activity is regulated to control a wide range of fundamental cellular processes including protein synthesis, mitochondria metabolism and autophagy. mTOR activation involves the tuberous sclerosis complexes (TSC) and Ras homolog enriched in brain (Rheb), in which Rheb acts downstream of TSC2 and upstream of mTOR. Biochemically, TSC2 is a GTPase-activating protein (GAP) and acts as a negative regulator of Rheb. Rheb is a small GTPase and binds to the kinase domain of mTOR to stimulate the phosphorylation and activation of mTOR in a GTP-dependent manner. In the signaling cascade, TSC2 serves as a convergence point for upstream signaling inputs to mTOR complexes 1 (mTORC1), in which phosphorylation and subsequent inactivation of TSC2 leads to activation of Rheb and subsequently activation of mTOR. mTOR interacts with other factors and serves as a core component of mTORC1 and mTORC2. In particular, ribosomal protein S6 kinases (S6Ks) and eIF4E-binding proteins (4E-BPs) represent the best-characterized downstream targets of mTORC1 whereby subsequent activation of ribosomal protein S6 and translation initiation factor eIF4E leads to up-regulation of protein synthesis.

In our previous studies, we uncovered a critical role for mTOR signaling in entrainment and synchronization of the SCN circadian clock[20,21,22]. First, light pulses at night activate the mTORC1/S6K pathway in the SCN, which causes phase shifts in behavioral rhythms and facilitates photic entrainment[20]. Second, the mTOR/4E-BP1 pathway controls mRNA translation of vasoactive intestinal polypeptide (VIP), a major neuropeptide responsible for neuronal synchronization in the SCN[21,22]. Further, genetic alteration of mTOR and its downstream signaling components impacted circadian rhythms in *Drosophila*[23,24] and in mice[21,22]. Interestingly, mTOR activities exhibit circadian rhythms in a variety of tissues and cells outside the central clock, including cardiac and skeletal muscles, adipocytes, retinal photoreceptors and renal carcinoma cells[25–29]. Inspired by these findings, in the present study, we employed genetic and pharmacological approaches to systematically examine the effect of mTOR perturbation on the autonomous properties of the circadian clock, including period length and rhythm amplitude, in central and peripheral circadian oscillators. We found that mTOR activation accelerates the speed and enhances the robustness of circadian oscillations in cellular clock models, tissues including the liver and the SCN, as well as in whole animals. As mTOR activity is tightly coupled to the nutrient and energy status in cells, our results indicate that cellular metabolism can impact circadian timekeeping via the mTOR pathway.

## Results

### *mTor* knockdown via RNAi lengthens circadian period length in cellular clock models

**Fig 1:**
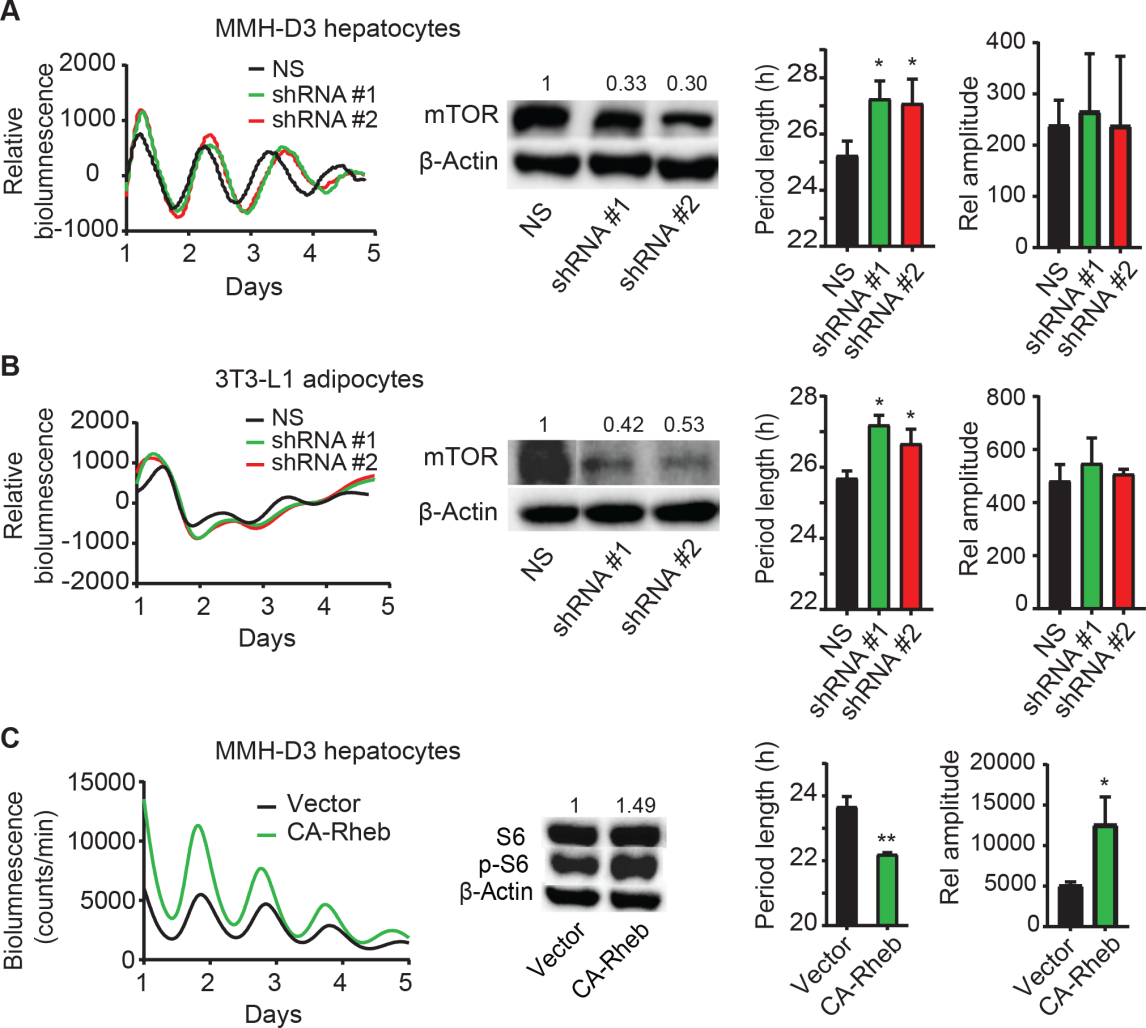
Genetic manipulation of mTOR pathway alters circadian clock function. RNAi knockdown of *mTor* lengthens the period length of circadian bioluminescence rhythms in MMH-D3 hepatocytes. (**A**) and 3T3-L1 adipocytes (**B**). Hepatocytes and adipocytes harboring the *Per2-dLuc* reporter were infected with lentiviral non-specific (NS) shRNA or shRNA constructs against *mTor*. Left panel: real-time bioluminescence expression was recorded in a Lumicycle luminometer on 35-mm culture dishes and the bioluminescence data are representative of at least three independent experiments. Middle panel: *mTor* knockdown efficiency was determined by Western blot analysis (middle). Right panel: period length and rhythm amplitude are mean ± standard deviation (SD) (n = 3 independent dishes). * p < 0.05 vs. NS. **(C)** Elevated mTOR via constitutively active Rheb shortens circadian bioluminescence rhythms in MMH-D3 hepatocytes. Bioluminescence rhythms from MMH-D3 hepatocytes harboring the *Per2-dLuc* reporter and overexpressing either the empty vector or constitutively active Rheb (CA-Rheb). p-S6 as a proxy of mTOR activation was hyper-phosphorylated in cells expressing CA-Rheb relative to vector control cells, whereas S6 levels were similar in the two cell lines. * p < 0.05 vs. Vector; ** p < 0.01 vs. Vector.

In our RNAi functional genomic screen[12], we identified the human *mTOR* gene as a circadian modifier, whereby knockdown altered circadian rhythms in human U2OS cells. We aimed to determine whether mTOR has a similar modifier role across species and under different physiological contexts. For this study, we used lentiviral shRNA to knock down gene expression and leveraged our previously developed reporter cell lines: mouse MMH-D3 hepatocytes and 3T3-L1 adipocytes, each harboring a *Per2-dLuc* reporter in which the expression of a destabilized luciferase is under the control of the *Per2* promoter[30]. We generated two functional shRNAs that effectively knocked down *mTor*, as determined by Western blotting analysis, and altered circadian bioluminescence rhythms in both cell lines (Fig 1A and 1B, left and middle panels). Compared to non-specific control, *mTor* knockdown caused significantly longer circadian period length in both MMH-D3 and 3T3-L1 cells (Fig 1A and 1B, right panel). *mTor* knockdown did not cause drastic changes in rhythm amplitude. Together with results from U2OS cells, our data suggests a ubiquitous role for mTOR in cellular clock models.

### mTOR activation by constitutively active Rheb alters circadian rhythms in hepatocytes

To further support the role of mTOR signaling in clock regulation, we asked whether mTOR activation and inhibition cause opposite clock phenotypes. Rheb is the upstream activator of mTOR. Previous studies show that the Rheb-Q64L mutant is constitutively active in its ability to directly interact with mTOR and stimulate its kinase activity[31], designated as CA-Rheb. CA-Rheb expression in MMH-D3 cells caused S6 hyper-phosphorylation (Fig 1C), indicative of hyperactive mTOR. As expected from the effect of mTOR inhibition, cells expressing constitutively active Rheb and consequently hyperactive mTOR have shorter period length and higher amplitude, compared to empty vector control (Fig 1C). Thus, mTOR inhibition (Fig 1A and 1B) and activation (Fig 1C) have opposite period and amplitude phenotypes, supporting a critical role of mTOR in regulating normal clock function in cells.

### mTOR inhibitors alter circadian rhythms in hepatocytes

**Fig 2:**
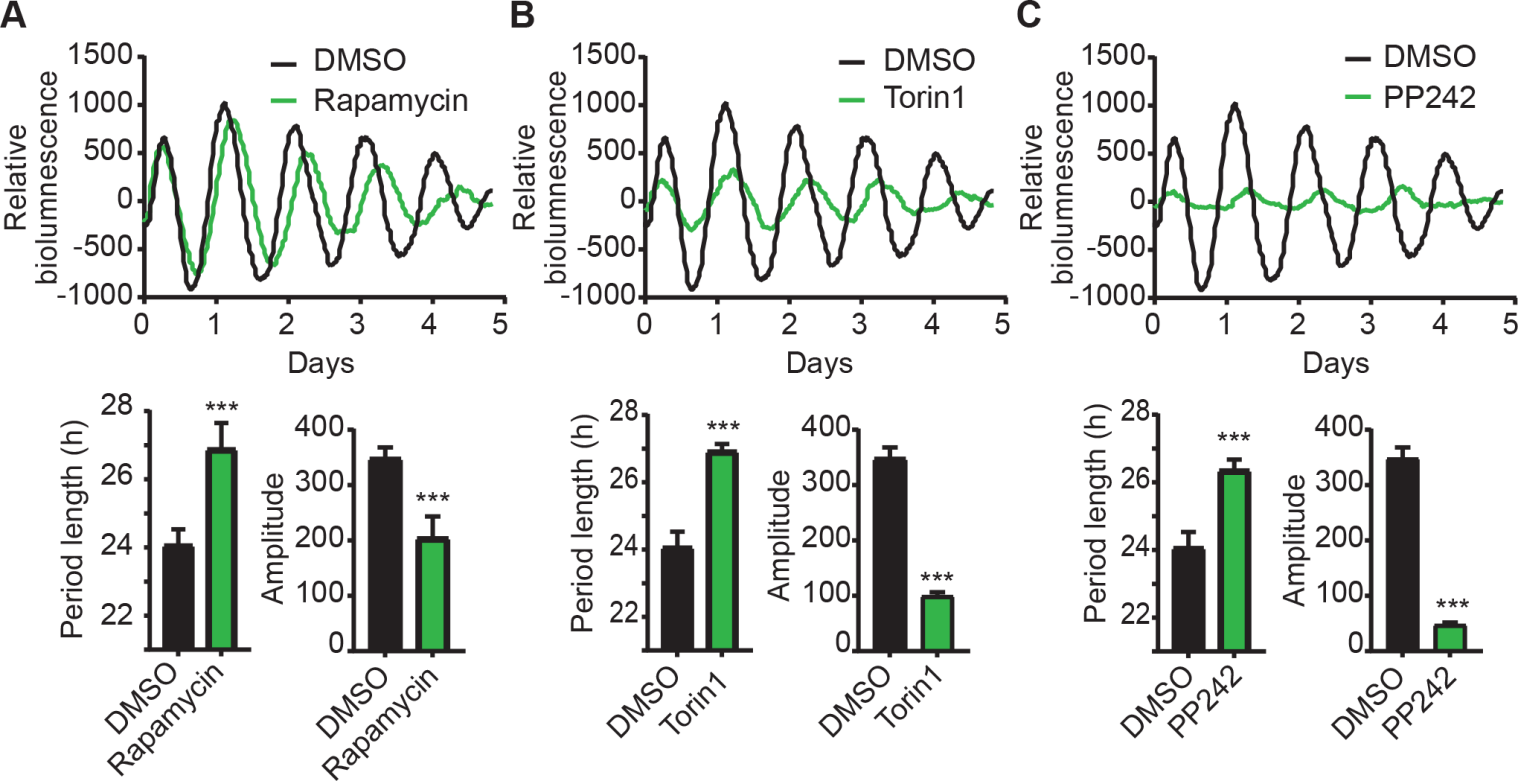
Pharmacological inhibition of mTOR alters circadian clock function in MMH-D3 hepatocytes. Top panel: representative records of bioluminescence rhythms of MMH-D3 hepatocytes harboring the *Per2-dLuc* reporter in the presence of mTOR inhibitors: 50 nM rapamycin **(A)**, 20 nM Torin1 **(B)**, or 10 uM PP242 **(C)**. Real-time bioluminescence expression was recorded in a Synergy microplate luminometer on 96-well plates. Bottom panel: period length and amplitude are mean ± SD (n = 8 independent wells) for each treatment. All three inhibitors caused significantly longer period length and lower rhythm amplitude. ***p < 0.001 vs. DMSO.

We reason that pharmacological inhibition of mTOR activity would have a similar phenotypic effect as RNAi knockdown. Thus, we tested the effects of rapamycin (or sirolimus), Torin1, and PP242 (or torkinib), three well-characterized mTOR inhibitors[32,33], in the MMH-D3 hepatocyte model. While rapamycin is a specific mTOR inhibitor toward mTORC1, Torin1 and PP242 target both mTORC1 and mTORC2. When incubated in the continuous presence of 50 nM rapamycin, MMH-D3 cells displayed significantly longer period length and lower amplitude, compared to DMSO control (Fig 2A). Consistent with rapamycin treatment, both Torin1 (20 nM) and PP242 (10 μM) also led to longer period lengths and reduced amplitudes (Fig 2B and 2C). Torin1 and PP242 led to stronger phenotypes than rapamycin, likely due to their different mechanisms of mTOR inhibition. Importantly, the effects of mTOR inhibition are not caused by decreased cell viability, as a medium change can reverse the effects. Given the strong inhibitory effect of these chemicals on amplitude, we suspect that the lack of an amplitude phenotype in RNAi experiments (Fig 1A and 1B) is likely due to relatively weak knockdown efficiency. Taken together, our data show that genetic knockdown and pharmacological inhibition caused similar clock phenotypes, which strongly support the role of mTOR in regulating autonomous circadian clock function.

### Constitutively active mTOR in *Tsc2*-deficient fibroblasts increases clock protein abundance

**Fig 3:**
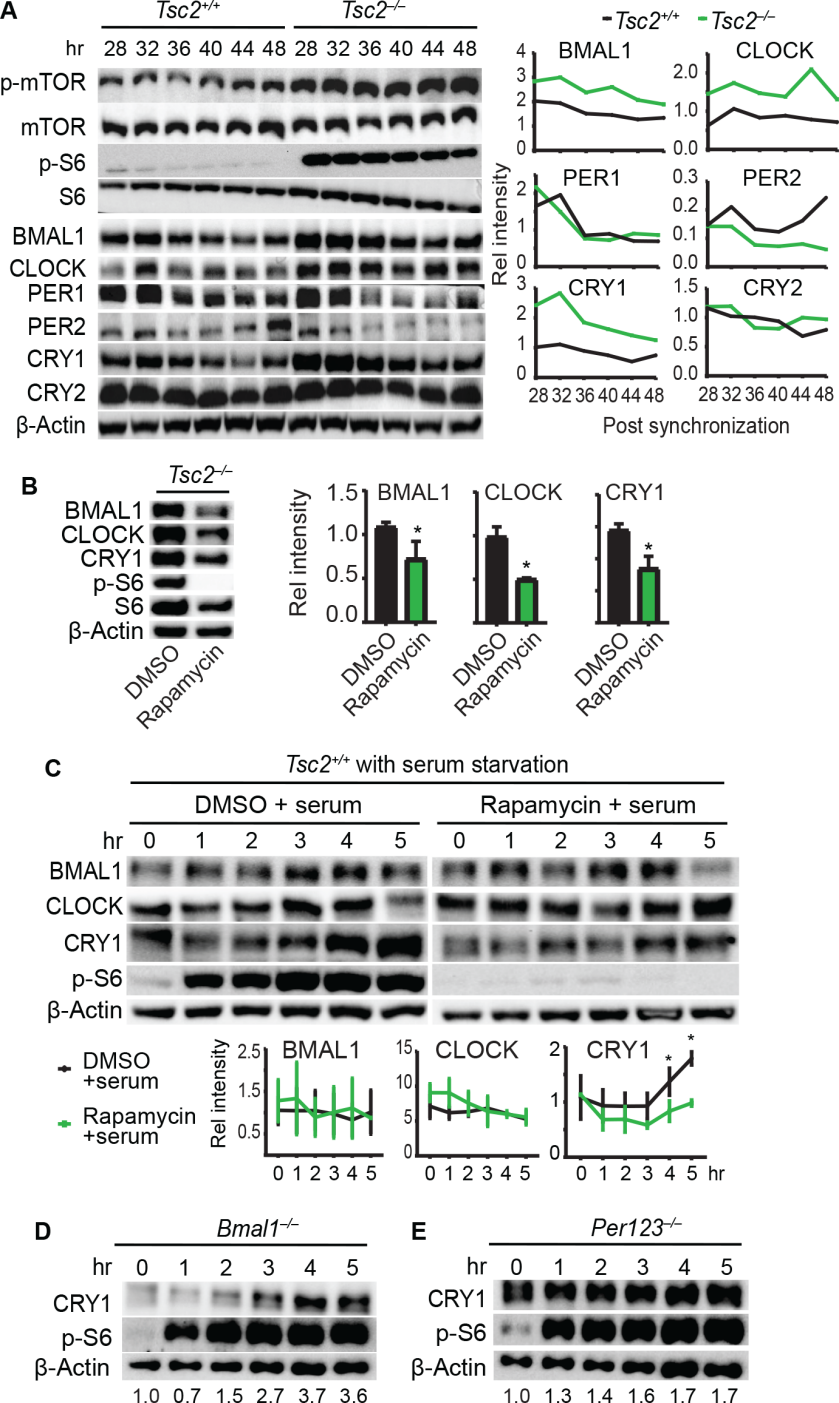
mTOR perturbation alters core clock protein expression in fibroblasts. **(A)** Western blots of *Tsc2*^*+/+*^ and *Tsc2*^*–/–*^mouse fibroblasts. Cells were synchronized with 200 nM dexamethasone (hr 0) and protein extracts were collected at 4-hr intervals starting at hr 28 post synchronization. Three independent dishes were collected for each time point and pooled for immunoblotting. Deletion of *Tsc2* led to constitutive activation of mTOR, as indicated by p-mTOR and p-S6 levels, and elevated steady-state levels of BMAL1, CLOCK and CRY1. Quantitation of the blots is shown to the right. **(B)** *Tsc2*^*–/–*^cells were treated with either DMSO or 10 nM rapamycin, and protein extracts were collected 4 hrs post treatment. Upon rapamycin treatment, the upregulated levels of BMAL1, CLOCK and CRY1 proteins were reduced. Quantitation of the blots is shown to the right. * p < 0.05 vs. DMSO. **(C)** WT fibroblasts were serum starved overnight, followed by addition of either DMSO or 10 nM rapamycin for 2 hrs (hr 0), and then the cells were stimulated with 50% serum. Cell lysates were collected at indicated times (hr 1–5). BMAL1, CLOCK and CRY1 were rapidly induced upon serum shock. However, unlike BMAL1 and CLOCK, CRY1 induction was compromised by rapamycin treatment. Data shown are representative of at least 3 independent experiments. Quantitation of the blots is shown at the bottom. * p < 0.05 vs. rapamycin. **(D-E)** *Bmal1*^*–/–*^(D) and *Per1/2/3*^*–/–*^fibroblasts (E) were starved overnight, followed by 50% serum treatment, as done in C. Deletion of *Bmal1* or *Per* genes did not significantly alter CRY1 induction by serum shock.

Given the strong cellular clock phenotypes presented here, and several previous studies that support the involvement of mTOR in clock gene expression[21,34,35], we determined the levels of canonical clock proteins in fibroblasts. For this, we leveraged the fibroblast model in which knockout cells are available. Because TSC2 is a negative regulator of Rheb and therefore mTOR, in *Tsc2*^*–/–*^fibroblasts (in *p53*^*–/–*^background), Rheb is constitutively activated, leading to hyperactive mTOR[36]. As a proxy of hyperactive mTOR, both mTOR and S6 were constitutively hyper-phosphorylated throughout the experiment, whereas their expression levels remained similar (Fig 3A). Interestingly, CRY1, BMAL1, and CLOCK levels were constantly elevated in *Tsc2*^*–/–*^cells throughout the experiment, as compared with the *Tsc2*^*+/+*^ cells. The increased protein levels in *Tsc2*^*–/–*^cells were reduced in the presence of rapamycin (Fig 3B), indicative of mTOR-dependent upregulation of CRY1, BMAL1, and CLOCK. However, we did not detect significant changes in the levels of PER1 and PER2 in the *Tsc2*^*–/–*^cells. Due to the limited duration of the experiment, circadian changes in mTOR activity and clock protein levels were not detected.

### CRY1 induction is dependent on mTOR but not on *Bmal1* or *Per* genes

The elevated clock proteins reflect their steady-state expression levels in *Tsc2*^*–/–*^cells where mTOR is constitutively activated. Similar to light-induced *Per1* and *Cry1* expression in the SCN[37,38], extracellular signals impinge on core clock genes to reset the molecular clock[39]. To evaluate the role of mTOR in inducing clock gene expression, we subjected the *Tsc2*^*+/+*^ cells to serum starvation for 16 hr and then treated the cells with 50% serum to stimulate mTOR activity, as reflected by the p-S6 levels (Fig 3C, left panel). We observed a rapid induction of CRY1, reaching the highest level at 5 hr following serum treatment. Treatment with rapamycin abolished serum-induced CRY1 upregulation (Fig 3C, right panel), suggesting that the CRY1 upregulation by serum is mediated, at least in part, by mTOR. In contrast, BMAL1 and CLOCK were not induced by serum stimulation and their levels were not reduced by rapamycin treatment in *Tsc2*^*+/+*^ cells, which could be due to relatively moderate effect of rapamycin.

Because the molecular clockwork consists of several temporally regulated negative and positive feedback loops, which complicates data interpretation, we asked whether CRY1 can be induced by serum in cells that lack a clock to intersect these feedback regulations. For this, we used fibroblasts deficient in *Bmal1*[40,41] and *Period* (*Per1*, *2*, *3*) genes[42]. We show that, following 50% serum shock, CRY1 was effectively induced in both *Bmal1*^*–/–*^and *Per1/2/3*^*–/–*^fibroblasts (Fig 3D and 3E). These data suggest that CRY1 induction mediated by mTOR signaling does not depend on the molecular clockwork, and more specifically, is independent on *Bmal1* or *Period* genes.

### Perturbation of mTOR activity alters the liver clock

**Fig 4:**
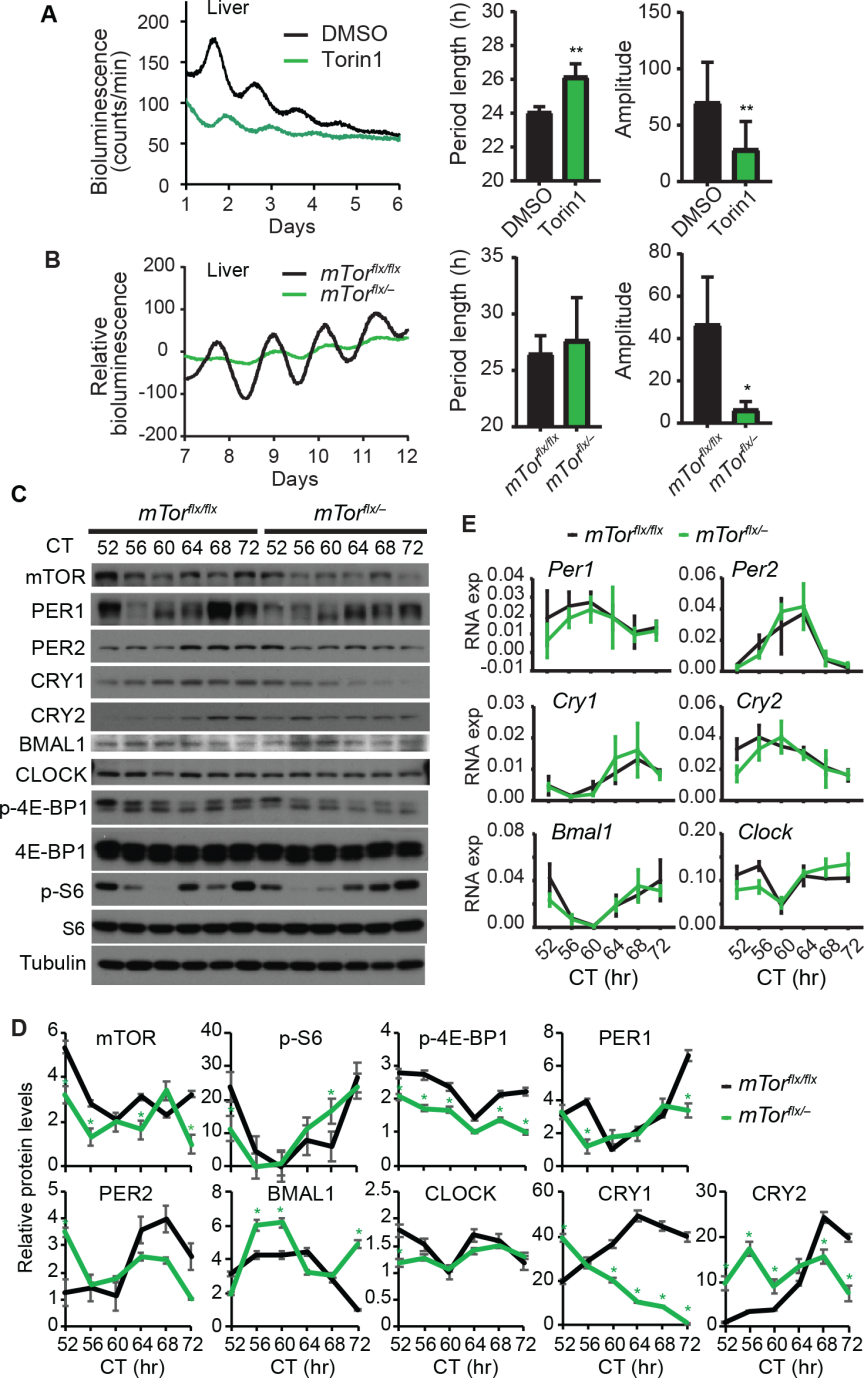
mTOR perturbation alters the liver clock function. **(A)** Bioluminescence rhythms of liver explants derived from *Per2*^*Luc*^ mice in the presence of DMSO or 20 nM Torin1. mTOR inhibition by Torin1 led to long period and low amplitude in liver explants cultured *ex vivo*. ** p < 0.01 vs. DMSO. **(B)** Bioluminescence rhythms of liver explants derived from *mTor*^*flxflx*^*;Per2*^*Luc*^ (wt control) and *mTor*^*flx/–*^*;Per2*^*Luc*^ (*mTor* heterozygous) mice. Heterozygous deletion of *mTor* reduced the rhythm amplitude in liver explants. * p < 0.05 vs. *mTor*^*flxflx*^. **(C-E)** Western blots (C and D) and Q-PCR (E) of liver tissue samples from *mTor*^*flxflx*^ and *mTor*^*flx/–*^mice. Mice were entrained to regular light/dark cycles and then released to constant darkness (DD), followed by tissue harvest at 4-hr intervals beginning at 52 hr in DD (CT52). While individual tissue samples were used for Q-PCR analysis, tissues from 3~5 mice were pooled at each time point for Western blotting. Relative Q-PCR values are presented for each gene and error bars represent SD of expression levels from 3 mice. Circadian time (CT): hours after animal release to constant darkness. Quantitation of the blots is shown in (D). * p < 0.05 vs. *mTor*^*flxflx*^.

The crosstalk between mTOR and clock function in hepatocytes prompted us to ask whether it also plays a role in the liver clock. To this end, we dissected liver explants from PER2::LUC fusion (*Per2*^*Luc*^) mice, cultured them *ex vivo*, and treated them with mTOR inhibitor Torin1. Our data show that mTOR inhibition reduced rhythm amplitude and lengthened period length (Fig 4A). These results are consistent with data from hepatocytes and suggest that mTOR plays a regulatory role in the intrinsic properties of the liver clock.

We then determined the role of mTOR function in the liver *in vivo*. Due to early embryonic lethality associated with null mice, we utilized the *mTor*^*flx/–*^heterozygous mice that retain 50% mTOR expression and activity[21,43]. We crossed these mice with the *Per2*^*Luc*^ reporter line to obtain *mTor*^*flx/–*^*;Per2*^*Luc*^ mice. Compared with the *mTor*^*flx/flx*^*;Per2*^*Luc*^ littermates, liver explants of *mTor*^*flx/–*^*;Per2*^*Luc*^ mice have significantly decreased rhythm amplitude (Fig 4B). The period length was not significantly different between the two groups. However, it is noted that the marked amplitude reduction compromised data fitting for period length analysis. Nevertheless, these data are consistent with mTOR inhibitor treatment and support the role of mTOR in regulating the liver clock.

To complement the bioluminescence data, we determined the expression patterns of endogenous core clock proteins by Western blotting analysis. Mice were entrained under the regular light/dark cycle and then released to constant darkness. Liver tissue samples were collected during the third day in constant darkness (DD), starting from circadian time hour 52 (CT52) for a complete cycle. Compared to that in *mTor*^*flx/flx*^ mice, mTOR levels were noticeably decreased in *mTor*^*flx/–*^mice, and accordingly, the p-4E-BP1 levels were also lower in *mTor*^*flx/–*^mice (Fig 4C and 4D). p-S6 level in *mTor*^*flx/-*^ liver was significantly decreased only at CT52, but significantly increased at CT68 as compared to the *mTor*^*flx/flx*^ liver, which may be due to shifted phase of circadian oscillations. Importantly, while S6 and 4E-BP1 levels were constant over time, p-S6 and p-4E-BP1 levels were strongly circadian, suggesting rhythmic mTOR activity in the liver. These results are in line with previous observations about circadian rhythms of mTOR activities in the liver and SCN[7,44–48]. Notably, significant oscillations of clock proteins (PER1, PER2, CRY1, CRY2, BMAL1) were detected in the liver of *mTor*^*flx/flx*^ and *mTor*^*flx/–*^mice. There was an apparent phase shift in their peak levels between the two genotype groups. For example, PER1 reached a peak at CT68 in *mTor*^*flx/flx*^ mice but at CT64 in *mTor*^*flx/–*^mice. Overall, the changes in core clock protein levels were modest, which is due at least in part to *mTor* heterozygosity and the robustness of the circadian system. Among all the clock proteins examined, we noticed a striking reduction in CRY1 expression in *mTor*^*flx/–*^mice, compared to *mTor*^*flx/flx*^ control mice, especially during peak hours (CT64-72). These results are consistent with and in support of our findings from MMH-D3 hepatocytes and liver explants cultured *ex vivo*, all pointing to an important regulatory role of mTOR in circadian clock function.

To accompany the Western blotting data, we performed qPCR analysis to determine the expression patterns of the transcripts of the core clock genes. No significant transcript changes for these genes were detected between the two genotypes (Fig 4E). The *Cry1* levels were also not significantly increased in *mTor*^*flx/–*^mice, relative to *mTor*^*flx/flx*^ mice. These data suggest that *Cry1* regulation by mTOR is primarily at the posttranscriptional level.

### mTOR perturbation alters the SCN clock oscillations

**Fig 5:**
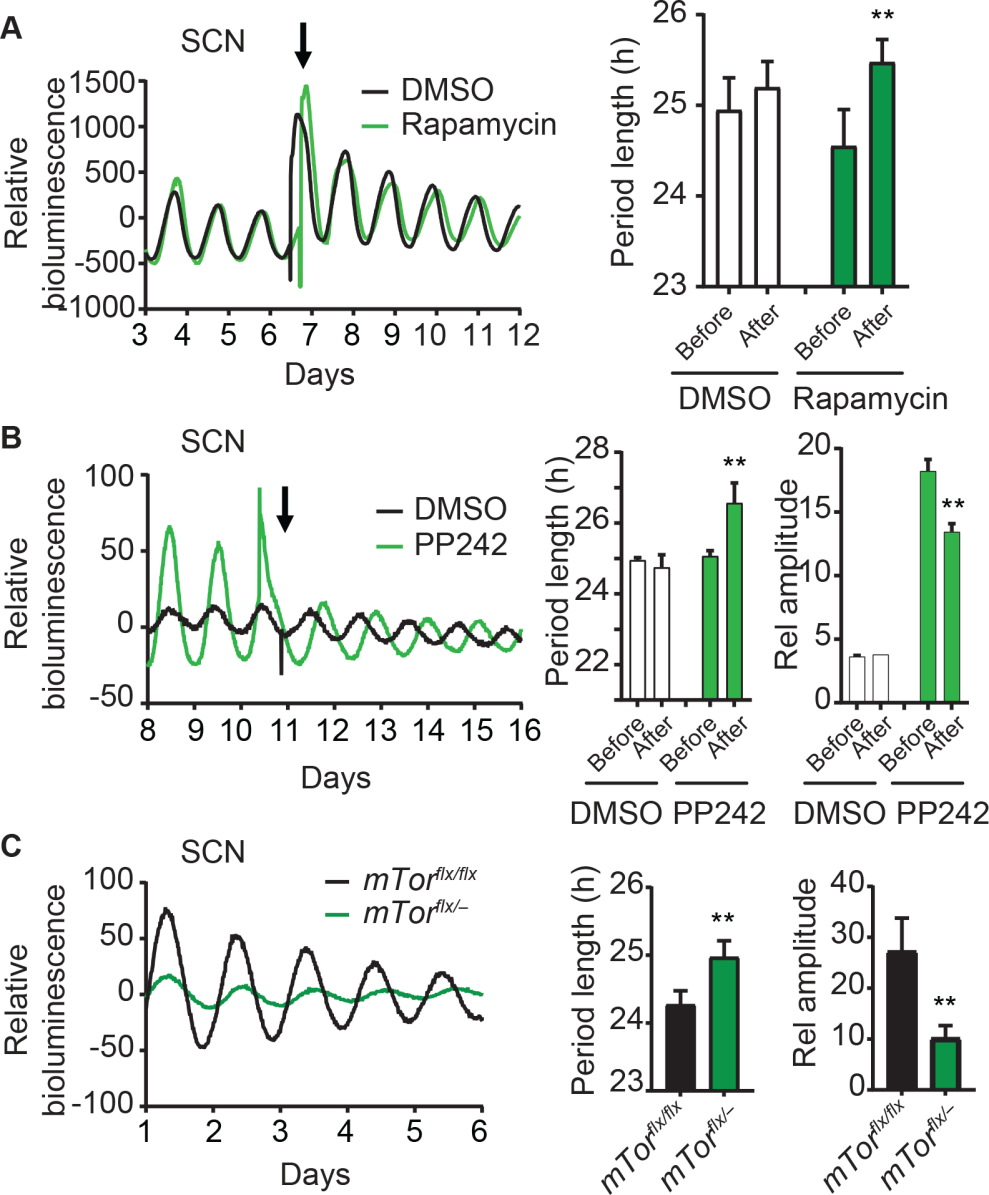
mTOR perturbation alters the central SCN clock function. **(A)** SCN explants from *Per2*^*Luc*^ reporter mice were treated with either DMSO or 50 nM rapamycin as indicated by an arrow. Representative bioluminescence records are shown (left). Period and amplitude data (right) are mean ± SD (n = 4 mouse SCN slices). ** p < 0.01 vs. rapamycin pre-treatment. Rapamycin treatment altered tissue-autonomous circadian rhythms of SCN explants cultured *ex vivo*. **(B)** Bioluminescence record from one representative SCN explant treated with PP242. Period and amplitude data (right) are mean ± SD (n = 4 mouse SCN slices). ** p < 0.01 vs. PP242 pre-treatment. **(C)** Bioluminescence rhythms of SCN explants derived from *mTor*^*flxflx*^*;Per2*^*Luc*^ and *mTor*^*flx/–*^*;Per2*^*Luc*^ mice. Heterozygous deletion of *mTor* reduced tissue-autonomous circadian rhythm amplitude and lengthened the period length of SCN explants. Period length and amplitude data are mean ± SD (n = 5 mouse SCN slices). ** p < 0.01 vs. *mTor*^*flxflx*^.

Our finding that mTOR regulates clock function in multiple peripheral cell/tissue models (U2OS, MMH-D3, 3T3-L1, and the liver) suggested a ubiquitous modifier role and raised the possibility that mTOR also regulates the central SCN clock function. Leveraging the mTOR inhibitors, we show that treatment of *Per2*^*Luc*^ SCN explants with rapamycin significantly lengthened the period length (Fig 5A). Similar to the inhibitory effect of rapamycin, PP242 also caused similar period lengthening effect in SCN explants and markedly decreased the amplitude (Fig 5B). Prompted by these observations, we asked whether genetic perturbation of *mTor* can alter the SCN clock. To this end, we show that SCN explants of *mTor* heterozygous *mTor*^*flx/–*^*;Per2*^*Luc*^ mice have significantly longer period lengths and lower amplitudes, compared to *mTor*^*flx/flx*^*;Per2*^*Luc*^ controls (Fig 5C). Taken together, our data suggest that mTOR functions not only in peripheral clock models but also in the central SCN clock.

### mTOR regulates circadian behavioral rhythms

**Fig 6:**
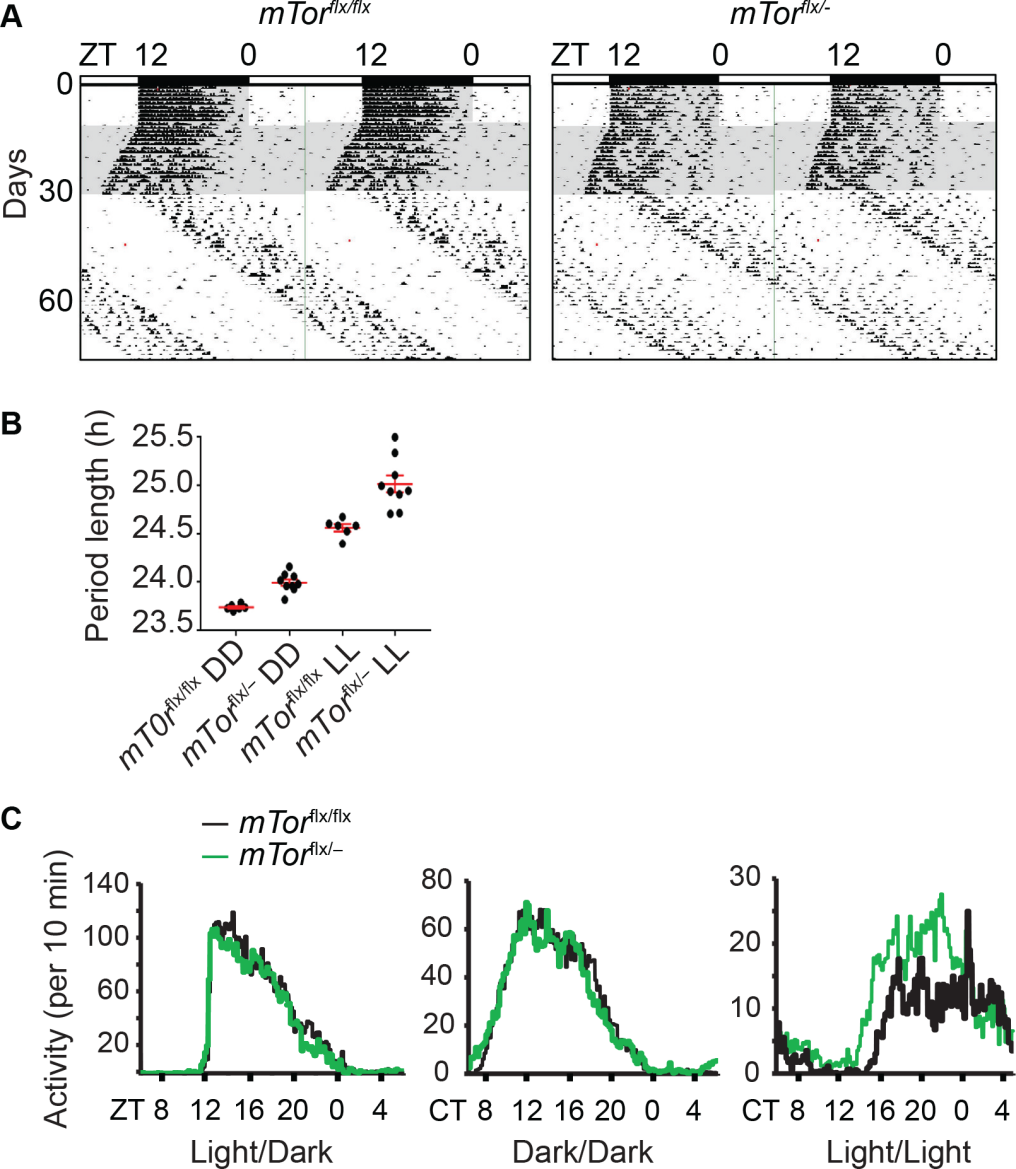
*mTor*^*flx/–*^mice have long period length of circadian locomotor activity rhythms. **(A)** Representative double-plotted actograms of wheel-running activity rhythms in *mTor*^*flxflx*^ and *mTor*^*flx/–*^mice. X axis: zeitgeber time (ZT) of the 12 h/12 h light/dark cycle (LD) indicated by the bar (top). Y axis: number of days during the experiment (left). Mice were first entrained to a regular LD cycle for 10 days and then released to constant darkness (DD) for 20 days. On the 31^st^ day, mice were released into constant light (LL) for 40 days. Grey and white areas indicate dark and light periods. **(B)** Circadian free-running period lengths of mice in DD and LL. Data are mean ± SEM (red) of individual values that are shown in black dots (n = 6 for *mTor*^*flxflx*^ mice and n = 9 for *mTor*^*flx/–*^mice. **(C)** Average wheel-running activity of mice expressed in wheel revolutions per 10 min across 24 h in LD, DD and LL.

The SCN clock generally reflects circadian animal behavior[4,49,50]. Our finding about mTOR function in the SCN prompted us to examine the effects of heterozygous *mTor* deletion on mouse circadian locomotor activity. As in SCN explants, we expected a similar period lengthening effect in *mTor* mutant mice. *mTor*^*flx/flx*^ and *mTor*^*flx/–*^mice were entrained to the standard 12h/12h light/dark (LD) cycle for 10 days and then released to constant darkness (DD). Mice were housed in wheel-running cages equipped to monitor their endogenous locomotor activity under free-running conditions. Like *mTor*^*flx/flx*^ mice, *mTor*^*flx/–*^mice were able to be entrained in the LD cycle and exhibited intact free-running rhythms in DD (Fig 6A). However, their circadian period was significantly longer in DD than their *mTor*^*flx/flx*^ littermates (Fig 6B; *mTor*^*flx/–*^: 24 hr ± 0.03, n = 9; *mTor*^*flx/flx*^: 23.74 hr ± 0.01, n = 6; p = 0.03, F = 4.24). Furthermore, activity offset was less precise in *mTor*^*flx/–*^mice and there was an apparent rhythm splitting that usually happens under constant light conditions[51,52], all pointing to an altered circadian behavioral rhythm and compromised synchrony of the SCN clock.

After mice were kept in DD for 20 days, they were released to constant light (LL) for 40 days. As in DD, *mTor*^*flx/–*^mice under LL also had significantly longer period length than their *mTor*^flx/flx^ littermates (Fig 6B; *mTor*^*flx/–*^: 25.02 hr ± 0.09, n = 9; *mTor*^*flx/flx*^: 24.57 hr ± 0.04, n = 6; p < 0.0001, F = 7.537). Interestingly, although the total activities were not different between WT and heterozygotes in LD and DD, *mTor*^*flx/–*^mice in LL did show more scattered locomotor activity, broader alpha (i.e. longer duration of active phase), and less precision in activity onset and offset, indicative of a compromised clock (Fig 6C). Taken together, our behavioral rhythm data are consistent with and in support of the reduced amplitude (Fig 6), compromised synchrony (Fig 6), and increased susceptibility to light-induced desynchrony of clock cells in the SCN of *mTor*^*flx/–*^mice[21].

## Discussion

It is well known that circadian clock regulates cellular metabolism. There has been considerable interest in studying the crosstalk between metabolism and the circadian clock[14]. However, it is not clear how various metabolic signals feed back into the clock mechanism. In the current study, we focused on the role of mTOR signaling in regulating the endogenous circadian clock function. Following our clock gene discovery through functional genomics, we employed an integrated approach combining genetic and pharmacological methods and examined the mTOR effects on circadian rhythms at multiple levels of biological organization, including fibroblasts, hepatocytes, adipocytes, the liver, the SCN, and the whole organism. We found that, while mTOR activation speeds up the clock oscillations (i.e. shorter period), mTOR inhibition lengthens circadian period. These phenotypes are consistent across multiple cell and tissue types including the central and peripheral oscillators. Moreover, mTOR also regulates rhythm amplitude: whereas mTOR inhibition dampens the amplitude, its activation increases the amplitude. Thus, certain level of mTOR activities appears to be required to maintain normal circadian oscillations in the SCN and peripheral oscillators.

The magnitude of the period changes in *mTor* heterozygous knockout mice (16 min in DD and 27 min in LL) was modest but significant, and is similar to that observed in other homozygous knockout mouse models, including *Clock* (20 min), *Per3* (30 min), *Nr1d1* (20 min), *Npas2* (12 min), and more recently, *Chrono* (18 min) ([10] and references cited therein). It should be noted that the behavioral phenotypes obtained in this study were from *mTor* heterozygous mice, due to lethality of homozygosity. Furthermore, the SCN clock generally reflects circadian animal behavior, and due to the robustness of the SCN clock, behavioral phenotypes are usually less dramatic than those in cellular clock models[4,49,50]. These data from the central SCN clock and peripheral oscillators strongly suggest that the mTOR pathway plays an important regulatory role in the mammalian circadian system.

Light is the most important and potent environmental cue to reset the circadian clock in the SCN. In our effort of searching for signaling pathways that couple photic cues to the SCN, we uncovered the role of the mTOR signaling in resetting or entraining the SCN and circadian behavior[20]. The mTOR activity in the SCN is highly circadian (high during day and low at night)[44], and light at night rapidly stimulates mTOR activation and signaling[53]. Inhibition of mTOR by rapamycin attenuates light-induced phase delay in mice[20]. Along this line, mTOR activities were later found to show daily rhythms in various tissues and cell types[7,25–29,45–48]. At the cellular level, circadian mTORC1 activity (as indicated by p-S6) closely correlates with circadian *Per1* expression in the brain[44], indicating that mTOR is important for regulating autonomous clock properties in the brain clock.

As mTOR is the key component in both mTORC1 and mTORC2, we targeted the mTOR gene to manipulate signaling of both complexes. However, as rapamycin and Rheb specifically regulate mTORC1[54,55], our results suggest that mTORC1 is a key signaling pathway that is linked to the clock machinery, consistent with findings in *Drosophila*[22]. However, contributions from mTORC2 cannot be excluded. Our results are also supported by a recent study showing that *Tsc2* mutant cells and mice have enhanced BMAL1 translation and short free-running periods[35], which is consistent with our findings in the current study (i.e. longer period in mTOR loss-of-function, and shorter period in gain-of-function). Intriguingly, however, these phenotypes in mice are opposite to that in *Drosophila*, where elevated and decreased TOR activity lengthened and shortened circadian behavioral rhythm period lengths, respectively[22,23]. In these studies, the authors studied the TOR effect on animal behavior, but not on cell or tissue models. We speculate that the phenotypic discrepancy could be due to differences between the two species, particularly at the behavioral level, where they have opposite anticipatory behavior associated with diurnality. Mechanistically, our data support the involvement of CRY1, BMAL1 and CLOCK in mediating the mTOR effect. However, unlike CRY1 in mammals which is the chief repressor that regulates period length, CRY in flies doesn't function as a repressor. This may explain the opposite phenotypic difference between the two species. Future studies of the mechanisms of the TOR effect in flies may help uncover the distinct, opposite phenotypes in the two different systems.

To gain mechanistic insight into the mTOR effect, we used *Tsc2*^*–/–*^cells, in which mTOR is constitutively activated. We found that core clock proteins including transcriptional activators BMAL1 and CLOCK and the chief repressor CRY1 were increased in *Tsc2*^*–/–*^cells. This upregulation is attributable at least in part to mTOR, because mTOR inhibition by rapamycin dramatically reduced their expression levels. Our finding is consistent with the recent study that showed that mTOR regulates BMAL1 translation, ubiquitination and degradation[35]. However, while both studies detected BMAL1 elevation upon mTOR activation, our data indicate that CRY1 induction through mTOR is independent on *Bmal1* or *Per1/2/3* (Fig 3). Our data support the notion that time-of-day-dependent mTOR activity regulates the magnitude of BMAL1, CLOCK, and CRY1 expression, thereby enhancing the amplitude of circadian oscillations. In particular, CRY1 appears to be highly responsive to acute mTOR activation, which then provides input to affect the clock function. The physiological importance of this observation remains to be investigated in future studies.

Several studies in recent years have linked the nutrient and metabolic states of an organism to the circadian clock[56]. These studies used genetic or feeding regime manipulations, including restricted feeding, calorie restriction, high fat diet, and genetic models of type 2 diabetes, and showed that changes in animal metabolic homeostasis alter the clock function in the SCN and peripheral tissues such as the liver and heart[57–61]. Recent studies have revealed several input mechanisms: AMP-activated protein kinase (AMPK) phosphorylates CRY1 for accelerated degradation[62], NAD^+^-dependent deacetylase sirtuin-1 (SIRT1) promotes BMAL1 and PER2 deacetylation[63–65], poly(ADP-ribose) polymerase (PARP1) ribosylates CLOCK[66], NADP^+^/NADPH ratio and oxidative stress regulates BMAL1/CLOCK activity that involves the redox-sensitive antioxidative transcription factor NRF2[67,68], hypoxia-inducible factor 1-alpha (HIF1α) regulates *Per2* transcription[69–71], and the tumor suppressor p53 modulates *Per2* both at the transcriptional and post-transcriptional levels[72,73]. Our work provides additional mechanistic details about how the mTOR pathway links metabolic signals to the circadian clock function.

It is well established that AMPK and mTOR serve as a signaling nexus for regulating cellular metabolism[74], and AMPK was shown to regulate CRY1 phosphorylation and degradation[62]. As AMPK is a negative regulator of mTOR, it’s likely that AMPK impacts the clock function by impinging upon the mTOR pathway. However, the precise mechanism of CRY1 regulation by mTOR is not clear and warrants detailed mechanistic studies. It is worth noting that, as mTOR resides at the center of a complex signaling network, manipulation of mTOR affects both the upstream and downstream events. In particular, several pathways converge on TSC1/2, and mTORC1 and 2 and their effectors have distinct but yet overlapping downstream effectors. As such, delineating the precise regulatory mechanisms of mTOR action will require much additional work in future studies. However, given that mTORC1 plays a critical role in specific protein translation and proteolysis, it is likely that differential protein synthesis and degradation, not at the transcriptional level, underlie the mTOR effects on BMAL1, CLOCK, and CRY1 proteins and on circadian oscillations.

Thus, data from this work and previous studies demonstrate that mTOR signaling is a multifaceted regulator of the circadian clock. First, it functions as part of the photic entrainment pathway to input to the SCN clock. Second, owing to its regulation by the clock, mTOR serves to provide rhythmic outputs from the clock to regulate circadian physiological and biochemical processes such as ribosomal biogenesis[46] and mRNA translation in the liver[48]. Further, mTOR also acts as a modifier to regulate the clock function. In peripheral tissue, especially the metabolically active liver, mTOR acts to modify the local clock in response to metabolic and physiological inputs. Intriguingly, as a nutrient/energy sensor, mTOR senses cellular nutrient and energy levels and integrates the inputs to the cells from upstream pathways mediated by insulin and growth factor receptor signaling. As such, the mTOR activity is regulated not only by the endogenous, anticipatory circadian mechanism, but also by extracellular signals (e.g. the light/dark cycles and nutrient availability). Thus, the mTOR pathway interacts with the circadian system in multiple tissues, and the interplay plays a key regulatory role in mammalian metabolism and physiology. Dysregulation of mTOR under pathological and diseases states such as obesity, diabetes and cancer, could have adverse effects on the circadian clock and circadian behavioral and physiological processes. As circadian clock dysfunctions are often identified in patients with metabolic syndromes, a better understanding of how metabolic signals are transduced to control cellular clock function will provide insights into pathogenesis of these diseases.

## Materials and methods

### Ethics statement

Animals were maintained in the animal facility at the University of Memphis or University of Minnesota Duluth. All animal experiments were conducted according to the National Institutes of Health Guide for the Care and Use of Laboratory Animals and approved by the Institutional Animal Care and Use Committee at University of Minnesota (No.1606-33864A) and the Institutional Animal Care and Use Committee at the University of Memphis (No. 0764).

### Animals

*mTor*^*flx/flx*^ *mice on a C57BL/6 background (kindly provided by Dr*. *Sara C*. *Kozma*, *University of Cincinnati and Dr*. *Nahum Sonenberg*, *McGill University) were crossed to CMV-Cre mice to generate mTor*^+/–^*mice[21,43], which were then crossed with mTor*^*flx/flx*^ *mice to produce mTor*^flx/–^*mice*. *The mTor*^*flx/–*^*line was crossed with mTor*^*flx/flx*^ *line to mTor*^flx/flx^ *(~50%) and mTor*^flx/–^*(~50%) mice used in the experiments*. *mTor*^flx/–^*mice were crossed with PER2*::*LUC (Per2*^*Luc*^*) reporter mice[75] to obtain mTor*^flx/flx^;*Per2*^*Luc*^ *and mTor*^flx/–^;*Per2*^*Luc*^ *mice*.

### Circadian behavioral assay

About 2 months old mice were individually housed in cages equipped with running wheels and locomotor activities were recorded as previously described[21,49,34]. Briefly, mice were entrained to a standard 12hr/12hr light/dark cycle for 10 days and then released to constant darkness (DD) for 20 days, followed by release to constant light (LL) for 40 days. Wheel-running activities were analyzed using ClockLab program (Actimetrics).

### Chemicals

Rapamycin, Torin1, PP242, and insulin were purchased from Sigma-Aldrich (St. Louis, MO) or Selleck Chemicals (Houston, TX).

### Lentiviral plasmid vector construction and viral production

For constructing lentiviral vectors expressing WT Rheb or constitutively active Rheb-Q64L mutant, two primers (forward: 5’-caccatggactacaaagaccatgacggt-3’; reverse: 5’-tcacatcaccgagcacgaagactttccttg-3’) were used to amplify the respective DNA fragments. The fragments were first sub-cloned into pENTR/D-TOPO vector (Invitrogen, Carlsbad, CA) and subsequently to the pLV7 destination vector that harbor a puromycin resistance gene via Gateway cloning to generate the pLV7-CMV-Rheb expression vector. Viral particles were generated following standard protocols in 293T cells, as described previously[76,77]. Viral particles were concentrated using Lenti-X concentrator (Clontech Lab, Mountain View, CA). Cells were infected and selected with puromycin to generate stable expression cell lines. For lentiviral shRNA vectors, we followed the procedures described in our previous study[30]. Non-specific shRNA construct (GCAACAAGATGAAGAGCAC) was described in that study. Five *mTor* targets were designed and tested for knockdown efficiency. The target sequences for knockdown in MMH-D3 cells were GTGGAGCCCTACAGGAAGT and GTGCTACATTGGCTGGTGT, and those for 3T3-L1 cells were GCCACACCGTGATGGAAGT and GTGCTACATTGGCTGGTGT. Viral particles were prepared as above and used to infect MMH-D3 cells. Two days post-infection, cells were selected with 2 μg/ml puromycin and stable cells lines were used for rhythm assays.

### Cell and tissue culture

Cell culture and growth conditions for fibroblasts, MMH-D3 hepatocyte and 3T3-L1 adipocytes were performed as previously described[30]. SCN and peripheral tissue slices were dissected and cultured in explant medium as described previously[49].

### Bioluminescence recording and data analysis

For real-time bioluminescence recording, we used a Lumicycle luminometer (Actimetrics) on 35-mm culture dishes and Synergy SL2 microplate reader (Bio Tek) on 96-well plates as previously described[30,49,76]. Lumicycle Analysis Program (Actimetrics) was used to analyze Lumicycle data to determine period length and rhythm amplitude. Briefly, raw data were fitted to a linear baseline, and the baseline-subtracted data were fitted to a sine wave (damped), from which period length and goodness of fit and damping constant were determined. For samples that showed persistent rhythms, goodness-of-fit of >80% was usually achieved. Due to high transient luminescence upon medium change, the first cycle was usually excluded from rhythm analysis. For amplitude analysis, raw data from day 3 to day 5 were fitted to a linear baseline, and the baseline-subtracted (polynomial number = 1) data were fitted to a sine wave, from which the amplitude was determined. Synergy luminometer data were analyzed with the MutiCycle Analysis program (Actimetrics), in which bioluminescence data were subtracted (first-order polynomial) and fit into a sine wave to determine circadian period length and rhythm amplitude.

### Western blot

Liver and cell lysates were prepared as previously described[78]. Briefly, liver tissue samples were homogenized with a pestle grinder and lysed in RIPA lysis buffer containing cocktails of proteases inhibitors (Roche) and phosphatase inhibitors (Sigma). Cells were harvested by trypsinization and immediately lysed in RIPA buffer. SDS-PAGE and Western blot analysis were performed as previously described[79]. The primary antibodies used in this experiment are as following: guinea pig antibodies against BMAL1, CLOCK, PER1, PER2, CRY1, CRY2 were from Choogon Lee’s lab; rabbit or mouse antibodies against mTOR, p-mTOR, S6, p-S6, 4E-BP1, p-4E-BP1 were purchased from Cell Signaling Technology (Danvers, MA); and CRY1, CRY2, PER2, Actin and Tubulin were from Santa Cruz Biotech. Horseradish peroxidase-conjugated secondary antibodies were used for all Western detection. ECL or SuperSignal West Pico substrate (Thermo Scientific) was used for chemiluminescent detection.

### Quantitative real-time PCR analysis

RNA extraction, reverse transcription, and quantitative real-time PCR were performed as previously described[30,49,34]. SYBR Green PCR master mix (Thermo Scientific) was used in qPCR. The primers used in qPCR analysis were described in the previous study[30].
